# Advances in Nanotechnology Development to Overcome Current Roadblocks in CAR-T Therapy for Solid Tumors

**DOI:** 10.3389/fimmu.2022.849759

**Published:** 2022-03-23

**Authors:** Juan Mi, Qing Ye, Yuanzeng Min

**Affiliations:** ^1^ Department of Pathology, The First Affiliated Hospital of University of Science and Technology of China (USTC), Division of Life Sciences and Medicine, University of Science and Technology of China, Hefei, China; ^2^ CAS Key Lab of Soft Matter Chemistry, University of Science and Technology of China, Hefei, China; ^3^ Department of Chemistry, University of Science and Technology of China, Hefei, China; ^4^ Department of Endocrinology, The First Affiliated Hospital of USTC, Anhui Provincial Hospital, University of Science and Technology of China, Hefei, China; ^5^ Hefei National Laboratory for Physical Science at the Microscale, University of Science and Technology of China, Hefei, China

**Keywords:** nanotechnology, CAR-T, solid tumor, immunity, therapeutic effect

## Abstract

Chimeric antigen receptor T cell (CAR-T) therapy for the treatment of hematologic tumors has achieved remarkable success, with five CAR-T therapies approved by the United States Food and Drug Administration. However, the efficacy of CAR-T therapy against solid tumors is not satisfactory. There are three existing hurdles in CAR-T cells for solid tumors. First, the lack of a universal CAR to recognize antigens at the site of solid tumors and the compact tumor structure make it difficult for CAR-T cells to locate in solid tumors. Second, soluble inhibitors and suppressive immune cells in the tumor microenvironment can inhibit or even inactivate T cells. Third, low survival and proliferation rates of CAR-T cells *in vivo* significantly influence the therapeutic effect. As an emerging method, nanotechnology has a great potential to enhance cell proliferation, activate T cells, and restarting the immune response. In this review, we discuss how nanotechnology can modify CAR-T cells through variable methods to improve the therapeutic effect of solid tumors.

## Introduction

CAR-T therapy has made remarkable achievements in the research and clinical treatment of cancer, especially in the treatment of B cell malignancies (1–3). Unlike conventional surgery, radiotherapy, chemotherapy, immune checkpoint blocking therapies, targeted drug therapy, and CAR-T cell therapies offer more therapeutic options for patients with previously refractory tumors (4–8). To date, the United States Food and Drug Administration has approved five CAR-T therapies, namely, -Kymriah, Yescarta, Tecartus, Breyanzi and Abecma, -for hematologic malignancies (9). However, CAR-T cell therapy has not achieved satisfactory results in the treatment of solid tumors, such as colon, kidney, and ovarian cancers, for which the best clinical trial outcome is stable disease (10–14).

To improve the efficacy of CAR-T therapy in solid tumors, CAR-T cells must overcome three obstacles. First, the lack of tumor-specific antigens, dense stroma and aberrant vasculature at the tumor site prevent CAR-T cells from efficiently targeting the solid tumor site (15). Second, the tumor immune microenvironment and immunosuppressive mechanisms reduce the antitumor activity of CAR-T cells in solid tumors. Finally, because of the initial differentiation state of selected T cells, the cumbersome production process of CAR-T cells, and the tumor microenvironment (TME) with low oxygen, acidity and nutrition, the survival and proliferation rates of CAR- T cells *in vivo* were low.

**Figure 1:**
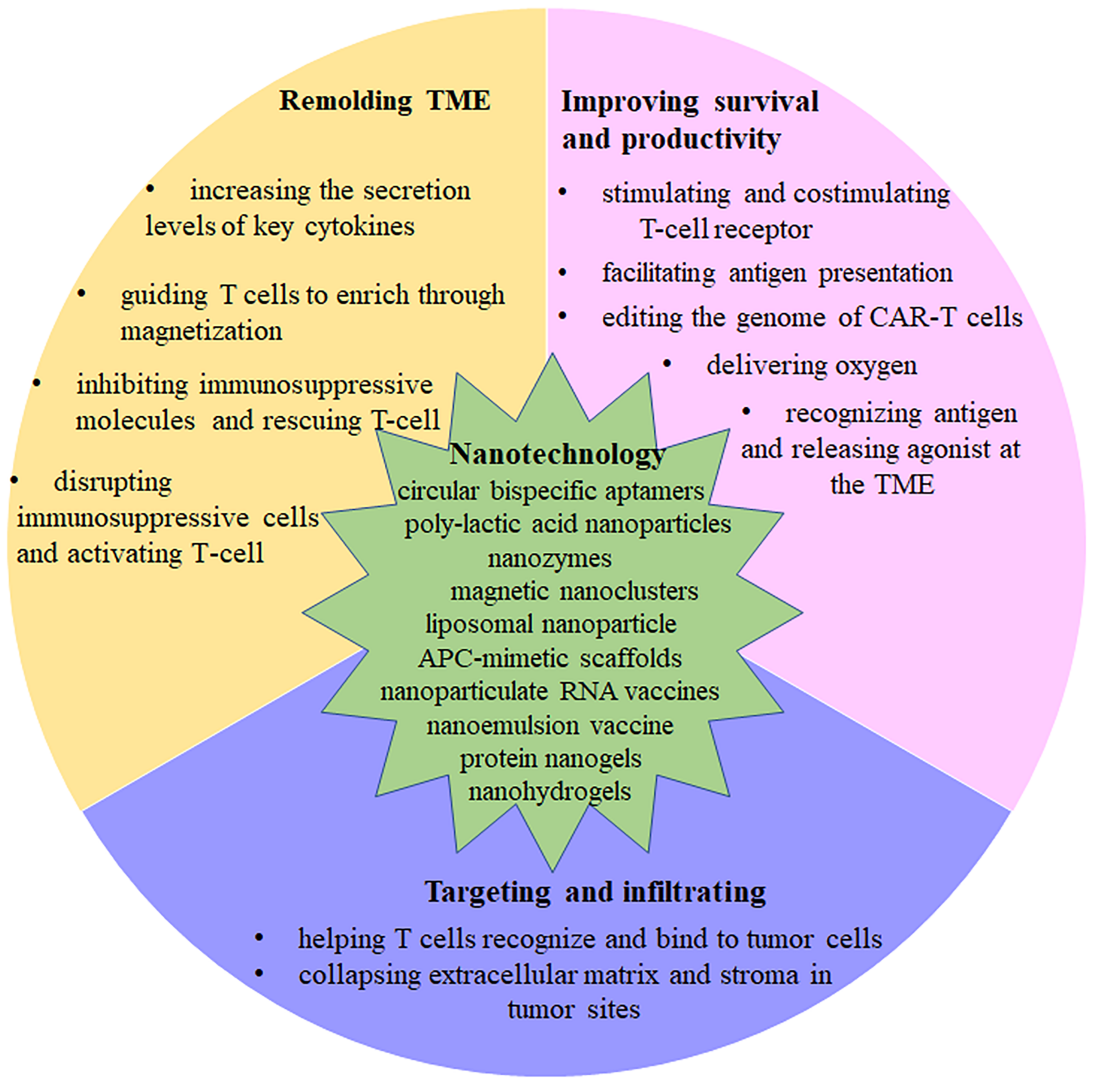
The mechanisms of Nanotechnology affect CAR-T function. Summary of strategies that are discussed in detail in this review.

Nanotechnology has multiple features that allow it to address the challenges of CAR T cell therapy in treating solid tumors. With optimal size, high surface area to volume ratio, a variety of shapes and components, as well as surface modification and charge, nanoparticles have a wide range of applications in tumor therapy (16–20). Nanoparticles employed in clinical treatments can be targeted to the site of the lesion with less accumulation in healthy tissue, stronger drug permeability, and retention, and can be rapidly biodegraded and eliminated without pharmacological and toxicological activities (21–23). Therefore, a number of researchers are exploring the use of nanoparticles in combination with CAR-T therapy to improve the efficacy of CAR-T therapy in solid tumors. Herein, we briefly introduce the three major challenges of CAR T cells in solid tumor therapy, and summarize how to combine nanoparticles with CAR T cells from different perspectives to solve the challenges in solid tumor therapy (**Figure 1**).

## Current Roadblocks in CAR-T Cell for Solid Tumors

Numerous clinical trials of CAR-T cell therapy for solid tumors have been carried out, and a meta-analysis of the efficacy of CAR-T therapy in solid tumors showed an overall response rate of 9%, although various therapeutic strategies have been implemented (24). There are three major factors that influence CAR-T therapy, as described below.

### Targeting and Infiltrating

CAR T cells are designed to select tumor-associated antigens (TAA) due to the lack of tumor-specific antigens (TSA). In a large number of clinical trials CAR T cell targeting tumor-associated antigens have been found cause damage to normal tissue with low expression of tumor-associated antigens during the process of recognizing and killing tumor cells, which is referred to as the off-target effect (25). Moreover, the reasons behind the success of CAR T cells in the treatment of hematologic tumors is that they can migrate in blood, lymph nodes, and bone marrow to interact with cancer cells (26). By dynamic imaging microscopy on fresh tumor slices from nine patients, Donnadieu et al. (27) investigated T cells with reduced motility in the stroma of human lung tumors, which hinted towards T cells facing difficulties in entering into the tumor due to the presence of obstacles. This makes it easy to understand that there are several other reasons why CAR T cells have difficulty entering solid tumor. Tumor-associated fibroblasts (TAFS) and abnormal vasculature at the tumor site result in compact tumor tissue and a dense extracellular matrix (ECM), which prevent CAR T cell to enter the solid tumor microenvironment (28, 29). The experiments conducted by Peschel et al. (30) confirm the lack of adoptively transferred T cells accumulation in solid tumors, while the infused HER2-specific T cells spread out in the breast cancer patient’s bone marrow. In addition, chemokines can induce T cell migration along the direction of increasing chemokine concentration. However, some solid tumors inhibit chemokine secretion and CAR T cells lack receptors that match chemokines secreted by solid tumors (31, 32), such that chemokine receptors on T cells mismatch with tumor secreted chemokines (33–35). Moreover, the low expression of adhesion molecules including ICAM-1 and 2, VCAM-1 and CD34 in tumor endothelial cells (EC) inhibit the effector T-cell from adhering to the EC and being transported to the tumor (36).

### Tumor Immunosuppression

Immunosuppression of the solid tumor microenvironment is another significant challenge for CAR-T therapy. The causes of tumor cells escaping the anti-tumor immune response are complex, including the presence of immunosuppressive cells, the presence of immunosuppressive cytokines and the absence of immune activating factors. The presence of immunosuppressive cells such as dendritic cells (DCs), myeloid-derived suppressor cells (MDSCs), regulatory cells (Tregs), and M2 macrophages in solid tumors sites, which secrete suppressive cytokines-such as transforming the growth factor-β (TGF-β), adenosine, interleukin-10 (IL-10), and vascular endothelial growth factor (VEGF) extracellularly-, suppresses the immune system and reduces the anti-tumor activity of CAR-T (37–40). Moreover, the immune checkpoint molecules PD-1 and CTLA4, when combined with the corresponding ligands, inhibit the killing effect of T cells on the tumor and the activation of T cells (41, 42).

### Survival and Proliferation

CAR T cells are targeted to the tumor site by a chimeric receptor mediated expressed on the T cell surface, and eliminate cancer cells through cell killing (43). Studies have shown that the long-term survival and proliferation of CAR T cells capable of maintaining normal function *in vivo* played a decisive role in the therapeutic effect (44). However, the expansion of the CAR T cells during the treatment of solid tumors is low *in vivo*. For example, Michael et al. detected a large number of CAR T cells in ovarian cancer patients after 2 days of transfusing *in vitro* gene-edited T cells back into the body, but the increase only lasted for about 1 month, and quickly declined to be virtually undetectable in the majority of patients (13). Even with large doses of CAR T cells, the presence of CAR T cells in the circulatory system was not detected (45). Moreover, clinical data showed that longer CAR-T cell persistence indicates longer delays, in the development of disease progression (46). The factors that influence the survival of CAR T cells in patients are complex, including the differentiation and functional status of CAR T cells, CAR target affinity, CAR immunogenicity, tedious time-consuming production process, immunosuppressive and hypoxic tumor microenvironment (47–49). Various nanotechnology strategies may improve CAR T cell persistence and expansion *in vivo*, which would endow CAR-T therapy with superior antitumor activity in the treatment of solid tumors.

## Application of Nanotechnology in CAR-T Therapy in Solid Tumors

### Nanotechnology to Aid CAR T Cell Target and Accumulate in Solid Tumors

To overcome the off-target effect caused by tumor-associated antigens, one group designed circular bispecific aptamers to help T cells recognize and bind to tumor cells. The aptamer can simultaneously bind naïve T cells and tumor cells, and then specifically activate T cells in the cell-cell junction complex. This strategy helps T cells pinpoint the tumor site and kill cancer cells. Thus, the targeted treatment of all kinds of cancer is possibly realized by the use of specific anticancer aptamers (50).

In an effort to arm CAR T cells to collapse physical barriers caused by angiogenesis, a dense extracellular matrix and stroma in tumor sites, researchers have proposed numerous of NP-based strategies (51, 52). By combing photothermal therapy with the adoptive transfer of CAR T cells, Gu et al. succeeded in promoting the accumulation and enhancing the conventional CAR-T therapy against solid tumors. The indocyanine green (ICG), a near-infrared (NIR) dye, is wrapped in poly(lactic-co-glycolic) acid (PLGA) nanoparticles. Once exposed to NIR light irradiation, ICG is used as the photothermal agent released into solid tumor (53–55). Mild hyperthermia of the tumor disrupts its compact structure, reduces interstitial fluid pressure (IFP), increases blood perfusion, and releases tumor-specific antigens that could significantly stimulate CAR T cells. After about 20 days, tumor growth was significantly inhibited, and no tumor cells were detected in about one-third of the treated mice (56). Other researchers fabricated indocyanine green nanoparticles (INPs) conjugated CAR T cells *via* the biorthogonal reaction. After mild photothermal intervention, tumor vessels expanded, blood perfusion increased, the ECM ablated and the tumor tissues became loose. Thus, INPs engineered CAR-T biohybrids accumulated and infiltrated extensively in the tumor, remodeled the TME, restarted the immune response, and boosted the efficacy of CAR-T immunotherapy. This microenvironment photothermal-remodeling strategy provides a promising prospect for CAR-T therapy in solid tumors (57).

### Nanotechnology to Remold Tumor Microenvironment to Stimulate CAR T Cells

To reset immunosuppression of cancer environment and promote the activation of CAR T cells, Zhao and colleagues effectively combined the use of the nanozymes method. They synthesized a tumor-targeting HA@Cu_2−x_S-PEG (PHCN) nanozyme with photothermal and catalytic properties. After irradiation by a near-infrared laser, the tumor extracellular matrix is damaged by converting light energy into local heat (58–60). Moreover, the reactive oxygen species by nanocatalyzed tumor therapy increased the secretion levels of key cytokines, such as the interferon and tumor necrosis factor as well as tumor-specific antigens, thus activating the corresponding CAR T cells at the tumor site (61).

To surmount the obstacle of hostile microenvironment, researchers tend to combine CAR-T therapy with the use of cytokines and/or antibodies. However, one problem is that CAR T cells and cytokines/antibodies disperse preventing their accumulation in the tumor sites (62, 63). Therefore, Xie et al. used a pH-sensitive benzoic−imine bond and inverse electron demand Diels−Alder cycloaddition to link magnetic nanoclusters (NCs) and the PD-1 antibody (aP) together to form NC-Ap. The constructed NC-aP binds to effector T cells due to their PD-1expression. Magnetic resonance imaging (MRI) guided T cells and aP to enrich in solid tumors through magnetization. Because of the acidic tumor microenvironment, the aP is released after the benzoic−imine bond, and then hydrolyzed. Consequently, the adoptively transferred T cells and aP synergistically inhibit solid tumor growth with a few side effects (64).

One of immunosuppressive molecules that inhibits the immune function of CD4^+^ and CD8^+^ T cells is adenosine. On the surface of activated T cells, the A2a adenosine receptor (A2aR) expressed and trigged adenosine to accumulate outside the cell, which suppressed T-cell proliferation and inhibited IFN–γ secretion (65, 66). Thus, using nanotechnology to efficiently transport SCH-58261 (SCH), a small molecule inhibitor of A2aR, to CAR T cells in tumors is a promising method. According to their report, Wang et al. used CAR-T therapy and SCH–loaded cross-linked multilamellar liposomes (cMLV) together, which significantly inhibited the tumor growth and improved the survival of treatment groups, the tumor infiltration rate of T-cells, as well as the expression level of IFN–γ *in vivo*. Through rescuing tumor-residing T-cell hypofunction, this method augments CAR T-cell efficacy in solid tumors (67).

The presence of immunosuppressive molecules- such as CTLA-4 and PD-L1 is another important cause of tumor immunosuppression. They enable tumor cells to escape surveillance by inhibiting the activation of immune cells, namely the “immune escape” (68, 69). To reset the suppressive solid tumor microenvironment, inhibitors targeting checkpoint molecules (such as CTLA-4, PD-1 and PD-L1) and CAR-T therapy were used in combination (70, 71). The disadvantages of using immune-checkpoint inhibitors (ICIs) include the emergence of a series of new immune-related adverse events and systemic toxicities (72). Stephan et al. designed a liposomal drug-loaded nanoparticle and decorated it with the tumor-targeting peptide iRGD. In addition, PI-3065, a PI3K kinase inhibitor that disrupts the function of immune-suppressive regulatory T cell subsets and myeloid-derived suppressor cell (40), and 7DW8-5, an immunostimulant-invariant natural killer T cell (iNKT) agonist was placed in the liposome (73, 74). They demonstrated that this new target nanoparticle alters the tumor immunosuppression and evidently enhances the anti-tumor activity of CAR T cells (75).

### Nanotechnology to Aid CAR T Cells Survive and Proliferate

The number of tumor-infiltrating lymphocytes is positively related with clinical outcomes of CAR-T therapies (36, 76, 77). T cells obtained from patients are limited, such that amplification *in vitro* may be an effective solution. In the body, the expansion of T cells requires the assistance of antigen-presenting cells (APC), which cannot be achieved *in vitro*. In light of this problem, Mooney et al. utilized mesoporous silica to create micro-rods and added in the APC-secreting factor interleukin-2, which extends the lifespan of T cells. They also coated the high-aspect ratio mesoporous silica micro-rods (MSRs) with supported lipid bilayers (SLBs) and a variety of antibodies that activate T cells, mimicking APC’s cell membrane. In cell culture, these rods randomly and automatically form a scaffold structure that allows T cells to move around and expand freely. Results showed that APC-mimetic scaffolds generate more CAR T cells and maintain good killing efficacy compared to conventional expansion systems (78).

The lack of proliferation signals in TME results in a low survival rate of CAR T cells. As emerging therapies, nanoparticulate RNA vaccines deliver liposomal antigen-encoding RNA (RNA-LPX) to activate T cells in cancer patients (79). Recently, Sahin et al. combined CAR-T with the nanoparticulate RNA vaccine to achieve the regulated proliferation of CAR-T cell expansion depending on RNA-LPX dose. The mechanism involves that antigen delivery to antigen-presenting cells in the spleen, lymph nodes, and bone marrow by intravenous injection, followed by the initiation of a toll-like receptor-dependent type-I IFN-driven immune-stimulatory program (80). Moreover, Chan et al. used the tailored nanoemulsion (Clec9A-TNE) vaccine to effectively solve the problem of limited antigen presentation, promote the proliferation of CAR T cells *in vivo*, and augment the efficacy of solid tumor therapy (81).

Conventional manufacturing of CAR-T cells includes several elaborate procedures such as isolation, modification and expansion, resulting a few effective redirected T cells that can be used. Meanwhile, virus transfection and electroporation are commonly used to help T-cells express targeted chimeric antigen receptors (CARs) or T cell receptors. In turn, these methods have drawbacks as they are time-consuming, have a small application scale (82, 83). Stephan et al. designed a new genetic programming named “hit-and run”, which transports mRNA nanocarriers into cells through simple mixing and transient expression of the target gene. The mRNA nanocarrier has three prominent advantages: (i) lyophilized mRNA NPs can be used for each application that has no effect on its properties and efficacy. (ii) NP uptake and transfection efficiency did not differ whether T cells proliferated or not. (iii) Lymphocyte-targeted mRNA nanocarriers can edit the genome of CAR-T-cells without influencing on their function. The paramount of this method is that it can simply produce CAR T cells at a clinical scale within a short time and without complex handling procedures *in vitro* (84).

Another novel method was developed to program numerous circulating T cells and effectively remove cancer cells *in situ*. On the surface of the biodegradable poly (β-aminoester)-based nanoparticles, anti-CD3e f(ab′)2 fragments are coupled with it to target T cells. Inside of the nanoparticles, the poly(beta-aminoester) (PBAE) polymer is assembled with microtubule-associated sequences (MTAS) and nuclear localization signals (NLS), which facilitates the gene transfer in the nucleus of the T cells. To maintain CAR expression in T cells, the CD19 CAR plasmid was flanked by the piggyBac transposase gene through a cut-and-paste mechanism. These stable polymer nanoparticles allow simple manufacture and storage, which provides a practical, economical and widely available pathway for CAR-T therapy (85).

The immunosuppression and hypoxia in the solid tumor microenvironment result in the weaking CAR T cells infiltration and proliferation. One research group constructed an injectable hydrogel-encapsulated porous immune-microchip system (i-G/MC) with oxygen reservoirs to intratumorally deliver CAR T cells. In the injectable i-G/MC system, IL-15-loaded alginate microspheres were made into thin immune-MCs (i-MCs), which were connected with HEMOXCell (Hemo; an oxygen carrier)-loaded alginate, and the alginate forms a gel layer by self-assembly (86). The i-MCs were highly porous and interconnected, which facilitates CAR T cell transport. Hemo, a marine extracellular hemoglobin, has a strong oxygen storage capacity and binds up to 156 oxygen molecules (per Hemo molecule). After the i-G/MC was injected into the solid tumor, the hydrogel (gel) layer degraded quickly, Hemo delivered oxygen to TME, as well as CAR T cells, and decreased the expression level of HIF-1α. Results showed that the immune-niche improves hypoxia TEM and promotes survival and infiltration of CAR T cells in solid tumors.

To avoid the side effects of systemically-administered supporting cytokines like interleukins, protein nanogels (NGs) with interleukin (IL)-15 super-agonist were designed. The NGs recognized the specific cell surface antigen and subsequently released the drug at the sites of antigen encounter, for instance, the tumor microenvironment. Most importantly, the NG delivery enhanced the cell proliferation level 16-fold in tumors and administered eight-fold higher doses of cytokine without toxicity (87).

## Conclusion

In preclinical studies, researchers have proposed a number of strategies to improve CAR T cell function through the use of nanotechnology. However, there are still some fundamental issues to be addressed in the clinical application of CAR T therapy. For example, the carcinogenicity, reproductive toxicity and persistence of magnetic nanoclusters are still unknown and therefore it cannot be used in clinical therapy. The use of near infrared laser will cause damage to human skin, short-term use will appear skin swelling phenomenon, long-term may affect human reproductive function and induce cancer. The safety, immunogenicity and toxicity of nano-vaccines have yet to be verified. Will nano-derivative biodegrades induce non-specific immune responses? Due to the specificity of tumor-associated antigens, the preparation cycle of tailored nanoemulsion vaccine is time consuming and involves high cost….

These questions from clinical studies may seem disappointing, but many studies have highlighted the potential of nanotechnology in combination with CAR T therapies for solid cancers, which giving us great hope for CAR T cells. Currently, there are about 40 CAR-T targets in clinical trials in solid tumors, which has significantly outnumbered hematological tumors. Different from CD19, which is often used as a target for CAR-T therapy in hematologic tumors, the main targets of CART development in solid tumors include Mesothelin, GD2, HER2, GPC3, Claudin18.2(CLDN18.2) and so on. Most CAR-T studies in solid tumors have low response rates in the 0-25% range (88). Recently, the EMA granted prime eligibility to CAR T - cell product candidate CT041, which against the claudin18.2 protein (CLDN18.2) for the treatment of gastric/gastroesophageal junction cancer. Results from a phase I clinical trial published in 2019 show a total objective response rate of 33% in a small group of patients with advanced gastric or pancreatic cancers, with no serious side effects (89). This means that CT041 is expected to become the world’s first approved solid tumor CAR T product, thus achieving zero breakthrough in solid tumor treatment.

## Author Contributions

JM: Conceptualization; writing-original draft. QY: Writing-review and editing. YM: Conceptualization; writing-review and editing. All authors contributed to manuscript revision, read, and approved the submitted version.

## Funding

New medicine grant from University of Science and Technology of China, Grant/Award Numbers: WK9110000103.

## Conflict of Interest

The authors declare that the research was conducted in the absence of any commercial or financial relationships that could be construed as a potential conflict of interest.

## Publisher’s Note

All claims expressed in this article are solely those of the authors and do not necessarily represent those of their affiliated organizations, or those of the publisher, the editors and the reviewers. Any product that may be evaluated in this article, or claim that may be made by its manufacturer, is not guaranteed or endorsed by the publisher.
